# Meta-Analysis of the Effects of Three-Dimensional Visualized Medical Techniques Hepatectomy for Liver Cancer with and without the Treatment of Sorafenib

**DOI:** 10.1155/2022/4507673

**Published:** 2022-09-13

**Authors:** Lei Zeng, Yuming Zhu, Peng Guo

**Affiliations:** Department of Hepatobiliary and Pancreatic Surgery, The Third Affiliated Hospital of Chongqing Medical University, Chongqing, China

## Abstract

**Background:**

The application of medical image three-dimensional (3D) reconstruction technology can provide intuitive 3D image data support for accurate preoperative evaluation, surgical planning, and operation safety. However, there is still a lack of high-quality evidence to support whether 3D reconstruction technology is more advantageous in liver resection. Therefore, this study systematically evaluated the clinical effects of 3D reconstruction and two-dimensional (2D) image-assisted hepatectomy.

**Methods:**

Databases were searched to collect published clinical studies on 3D reconstruction technology and 2D image-assisted liver resection. Data were extracted from the database construction to March 2022 and the risk of bias in the included studies was evaluated. Meta-analysis was performed using RevMan5.3 software.

**Results:**

A total of 13 clinical studies were included, including 1616 patients, 795 in the 2D group and 819 in the 3D group. The meta-analysis showed that the incidence of postoperative complications was lower in the 3D group than in the 2D group (OR = 0.64, 95% CI = 0.49–0.83, *P*=0.001) and also reduced operation time (SMD = −0.51, 95% CI = −0.74∼−0.27, *P* < 0.0001), decreased intraoperative blood loss (SMD = −63.85, 95% CI = −98.66–29.04, *P*=0.0003), decreased incidence of postoperative liver failure (OR = 2.42, 95% CI = 0.99–5.95, *P*=0.05), decreased postoperative recurrence rate (OR = 0.29, 95% CI = 0.16–0.53, *P* < 0.0001), and increased postoperative survival rate (OR = 2.19, 95% CI = 1.49–3.23, *P* < 0.0001).

**Conclusions:**

Current data suggest that 3D reconstruction-assisted hepatectomy can reduce intraoperative blood loss, postoperative complications, and recurrence, and improve postoperative survival. Therefore, the 3D reconstruction technique is worthy of application and promotion in assisted liver resection.

## 1. Introduction

The liver is the largest organ in the human viscera and the largest digestive gland in the human digestive system [1–3]. At the same time, it is also one of the tumor-prone sites. Pathological examination still plays an important role in guiding clinical treatment and evaluation as well as disease prognosis, especially in the diagnosis of different liver diseases [4]. Therefore, a biopsy is still the criterion for the diagnosis of benign and malignant liver lesions, but the biopsy cannot comprehensively evaluate the information of lesions. Imaging technology reduces the need for liver biopsy to a certain extent [5]. Its blood supply system has two pipeline systems: the portal vein and the hepatic artery. The portal vein supplies about 75% of the blood volume and about 50% of the oxygen, and the hepatic artery supplies about 25% of the blood volume and 50% of the oxygen [6]. Due to the variable anatomical structure of liver development, the variation rate of the hepatic arteries is very high. At present, most clinical classification standards used are Michels [7] type 10 or Hitta [8] type 6. The portal vein is generally divided into five types of variation [9], and its variation type is less than that of the hepatic artery, mainly because the portal vein system is composed of superior mesenteric vein, a splenic vein, etc., and then branches in the liver, starting with capillary network and ending with a capillary network. In addition, in hepatectomy, due to a large number of hepatic duct systems and interlaced multi-stage duct branches, liver surgery becomes more difficult and the importance of preoperative comprehensive evaluation becomes more prominent [10]. Traditionally, clinical surgeons used color Doppler ultrasound, CT, MRI, and other related auxiliary examinations two-dimensional (2D) to perform preoperative anatomical positioning [11]. Doctors diagnose and make preoperative plans relying on experience and a 2D medical image of the patient, which is highly subjective and uncertain [12]. With the rapid development of digital technology, imaging is combined with clinical application to achieve a more accurate curative effect. The three-dimensional (3D) reconstruction technique is to establish mathematical models suitable for computer expression and processing of 3D bodies [13]. We process, analyze, and reconstruct it in the computer environment, and establish the key technology of virtual reality expressing the objective world in the computer. The clinical application of three-dimensional reconstruction technology is to reconstruct a 3D image model of image data. Compared with 2D images such as CT and MRI, 3D reconstruction images have a dimensional feeling and at the same time, more accurately describe the relationship between the tissue lesion location and organs, and the reconstruction of 3D viscera can be performed separately according to the requirements of the performer: artery imaging, single vein imaging, space rotation, viscera blur, simulated surgical resection, and residual organ volume evaluation [14–16]. Using 3D reconstruction technology can get clear, intuitive 3D graphics, which can clearly reflect the liver, lesions, venous system, arterial system, and bile duct system. The morphological characteristics and 3D anatomical structure of liver lesions are clear. The portal vein classification and hepatic artery type can be intuitively displayed.

However, there is still a lack of high-quality evidence to support whether the 3D reconstruction technique is more advantageous in liver resection. Therefore, this study systematically evaluated the clinical effect of using 3D reconstruction technology and 2D imaging to compare the clinical effect of assisted hepatectomy and provided a reference for the selection of a clinical treatment plan.

## 2. Materials and Methods

### 2.1. Inclusion Criteria

Study type: clinical study on the application of the 3D reconstruction technique in hepatectomy. Randomized controlled trials (RCTS) were the first choice. If RCTS were not available, other types of clinical controlled trials were included. Participants: patients who have been clinically diagnosed with primary benign or malignant liver tumors, hepatolithiasis, metastatic liver tumors, or other diseases requiring surgical treatment. Intervention and control: the liver of the experimental group was evaluated by the 3D reconstruction technique before surgery, and the liver of the control group was evaluated by conventional 2D imaging before surgery. Outcomes: (1) postoperative complications; (2) operating time; (3) intraoperative blood loss; (4) the incidence of liver failure; (5) the postoperative 1-year recurrence rates; and (6) the postoperative 1-year survival rates.

### 2.2. Exclusion Criteria

The exclusion criteria were as follows: (1) repeated literature; (2) non-English literature; (3) the original research data cannot be extracted; (4) inconsistent intervention measures; and (5) the control group (2D group) was not set in the experimental design.

### 2.3. Literature Retrieval Strategy

Embase, PubMed, and the Cochrane Library databases were searched for published clinical trials on 3D reconstruction compared with 2D image-assisted liver resection from database establishment to March 2022. Search terms: 3D; 3D visualization reconstruction; hepatectomy; and hepatectomies.

### 2.4. Literature Screening and Data Extraction

First, the repeatedly published literature was screened out, and then, the literature was screened by reading the title. After excluding clearly irrelevant literature, the abstract and the full text of the literature were further read, and finally, the inclusion criterion was determined. The main contents of data extraction include the following: (1) basic data, such as first author, year of publication, study time, type of study design, sample size, and type of disease studied. (2) Basic characteristics of participants, such as age, sex, and disease status. (3) Intervention, such as preoperative evaluation methods and postoperative actual measurement indicators. (4) Bias risk assessment. (5) Outcomes, including postoperative complication rate, intraoperative blood loss, postoperative liver failure rate, postoperative recurrence rate, and survival rate.

Risk assessment of bias in the included studies.

Since RCTS were not retrieved, the Newcastle–Ottawa Scale (NOS) [17] was used to evaluate the risk of bias in included studies.

### 2.5. Statistical Method

The standard mean difference (SMD) was used to measure continuous variables, the rate ratio (RR), and 95% confidence interval (CI) were used to measure categorical variables, and the funnel plot was used to evaluate publication bias. The Egger analysis was carried out with Stata 15.0. *P* < 0.05 was considered statistically significant.

## 3. Results

### 3.1. Literature Screening Results

**Figure 1:**
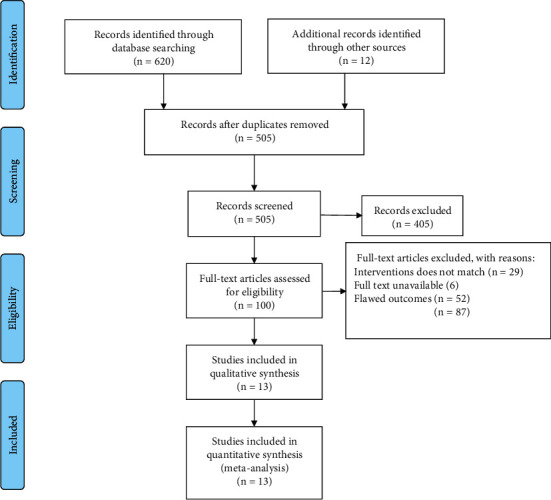
Flow diagram of the literature selection.

A total of 632 relevant literatures were obtained in the initial search and 13 clinical studies were finally included after screening. The literature screening process and results are shown in Figure 1.

### 3.2. Basic Features of the Included Studies

11 studies [18–28] reported the incidence of postoperative complications, 12 studies [18–22, 24–30] reported the duration of surgery, 4 studies [20–22, 25] reported the incidence of postoperative liver failure, 10 studies [18–21, 24, 25, 27–30] reported the amount of intraoperative bleeding, 5 studies [18, 21, 22, 24, 27] reported survival rates, and 4 studies [18, 21, 24, 29] reported recurrence rates. A total of 1616 patients were included, including 795 patients in the 2D group and 819 patients in the 3D group. The basic characteristics of the included study are shown in Table 1.

### 3.3. Meta-Analysis

#### 3.3.1. Postoperative Complications

**Figure 2:**
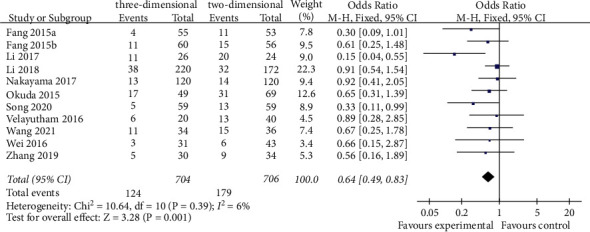
Forest plot of OR of comparison of postoperative complications between the three-dimensional group and the two-dimensional group.

A total of 11 studies reported postoperative complications [18–28]. The meta-analysis of the fixed-effect model showed that the incidence of postoperative complications in the 3D group was lower than that in the 2D group (OR = 0.64, 95% CI = 0.49–0.83, *P*=0.001), and the difference between the two groups was statistically significant (Figure 2).

#### 3.3.2. Operating Time

**Figure 3:**
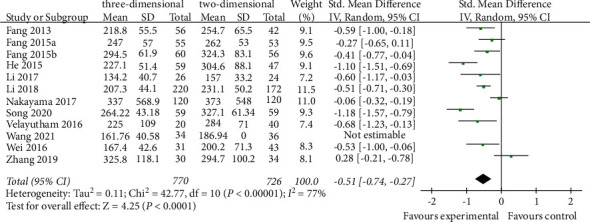
Forest plot of SMD of comparison of operating time between the three-dimensional group and the two-dimensional group.

Operating time was reported in 12 studies [18–22, 24–30]. The meta-analysis of the random effects model showed that the operation time of the 3d group was less than that of the 2D group (SMD = −0.51, 95% CI = −0.74∼−0.27, *P* < 0.0001), and the difference between the two groups was statistically significant (Figure 3).

#### 3.3.3. Intraoperative Blood Loss

**Figure 4:**
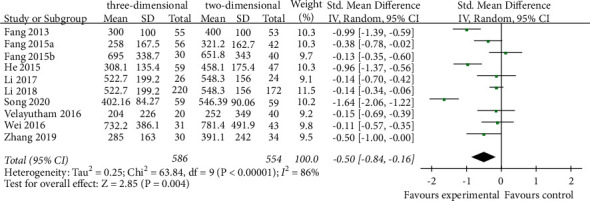
Forest plot of SMD of comparison of intraoperative blood loss between the three-dimensional group and the two-dimensional group.

A total of 10 studies reported intraoperative blood loss [18–21, 24, 25, 27–30]. The meta-analysis of random effects model showed that intraoperative blood loss in the 3D group was less than that in the 2D group (SMD = −63.85, 95% CI = −98.66–29.04, *P*=0.0003), and the difference between the two groups was statistically significant (Figure 4).

#### 3.3.4. The Incidence of Liver Failure

**Figure 5:**
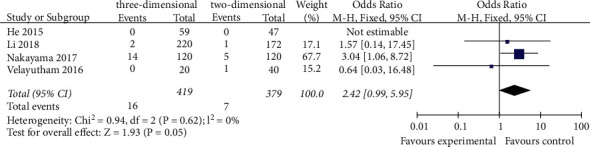
Forest plot of OR of comparison of the incidence of postoperative liver failure between the three-dimensional group and the two-dimensional group.

The incidence of liver failure was reported in four studies [20–22, 25]. The meta-analysis of the fixed effects model showed that the incidence of postoperative liver failure in the 3D group was lower than that in the 2D group (OR = 2.42, 95% CI = 0.99–5.95, *P*=0.05), and there was no statistical significance between the two groups (Figure 5).

#### 3.3.5. The Postoperative 1-Year Recurrence Rates

**Figure 6:**
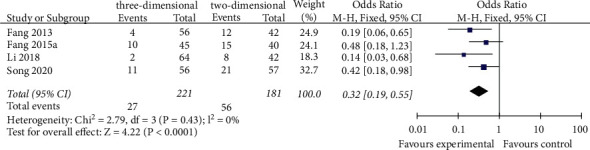
Forest plot of OR of comparison of the postoperative 1-year recurrence rates between the three-dimensional group and the two-dimensional group.

The 1-year recurrence rates were reported by four studies [18, 21, 24, 29]. The fixed effects model analysis showed that the rate of postoperative recurrence in the 3D group was lower than that in the 2D group (OR = 0.32, 95% CI = 0.19–0.55, *P* < 0.0001), and the difference between the two groups was statistically significant (Figure 6).

#### 3.3.6. The Postoperative 1-Year Survival Rates

**Figure 7:**
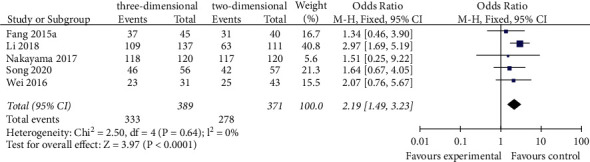
Forest plot of OR of comparison of the postoperative 1-year survival rates between the three-dimensional group and the two-dimensional group.

Five studies reported 1-year survival rates of the software [18, 21, 22, 24, 27]. The meta-analysis of fixed effects model showed that the postoperative survival rate in the 3D group was higher than that in the 2D group (OR = 2.19, 95% CI = 1.49–3.23, *P* < 0.0001), and the difference between the two groups was statistically significant (Figure 7).

#### 3.3.7. Sensitivity Analysis

RevMan 5.3 software was used to conduct a sensitivity analysis of outcome indicators by removing single included studies one by one, and the combined results did not change significantly, indicating that the meta-analysis results of this study were relatively stable.

#### 3.3.8. Publication Bias

**Figure 8:**
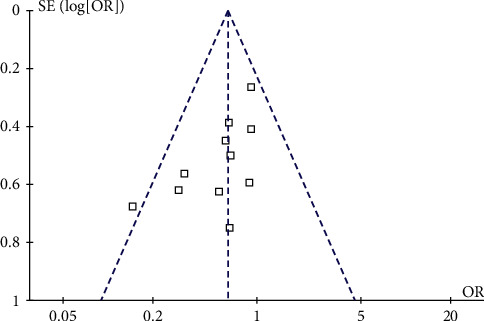
Funnel plot based on incidence of postoperative complication.

Based on the incidence index of postoperative complications, RevMan 5.3 software was used to draw a funnel plot for publication bias analysis, and the results showed that the distribution of each study on both sides of the funnel was not completely symmetrical, suggesting the possibility of publication bias (Figure 8).

## 4. Discussion

Since the 1970s, medical imaging equipment has been continuously improved, and there are more and more medical imaging methods. New imaging techniques provide a very effective means to observe the function of tissues and organs, thus becoming an important medical diagnostic tool. However, the 2D imaging is difficult to meet the requirements of accurate preoperative evaluation and preoperative planning, so it can only make a qualitative analysis of the disease, and the diagnosis result depends on the experience of reading film and subjective understanding of medical imaging. When different doctors diagnose the same disease, they sometimes come up with different results. After the 1990s, computer image processing technology developed rapidly and gradually penetrated the medical field. We use computers to analyze and process 2D images, segmentation, extraction, 3D reconstruction, and so on. With the gradual popularization and application of the 3D reconstruction technology in clinical practice, the technology has gradually transferred from orthopedic-assisted nail placement [31], craniocerebral model reconstruction in neurosurgery [32, 33], and plastic surgery [34–37] to the field of hepatobiliary surgery [38], which has become the preoperative diagnosis and evaluation of hepatobiliary diseases and the development of surgical planning [39].

On the 3D liver models, the virtual liver excision technique can accurately provide safe surgical removal and judge the distribution range of the blood vessels in advance. Then, we calculate the blood vessels and their branches' area and avoid the risk of liver parenchyma perfusion inadequacy or obstruction; the maximum limit retains the functional liver tissue. By observing virtual liver resection, doctors can evaluate and design the best surgical resection plan, so as to improve the success rate of complex liver resection, reduce postoperative complications, and accelerate the recovery of patients.

The results of this study show that, compared with traditional 2D imaging, 3D reconstruction can reduce postoperative complications and intraoperative blood loss, and the volume of liver resection predicted by precise planning is consistent with the actual volume of liver resection. It avoids the problems of liver insufficiency or even failure caused by the insufficient residual volume of the liver after the operation.

In this study, 12 studies were included and no RCTs were included, so study type-based subgroup analysis was not conducted. Differences in basic diseases, surgical methods, technical level, and study design of included patients will affect the reliability of the results.

In conclusion, the 3D liver reconstruction is helpful for safety assessment before anatomic hepatectomy and is beneficial for fine operation during surgery, reducing intraoperative blood loss, shortening treatment time, and reducing complications. However, due to the limitation of the quantity and quality of the included literature, more reliable conclusions from high-quality RCT studies are needed to better determine the practical application value of 3D reconstruction of medical images. However, current evidence shows that the application of the 3D reconstruction-assisted liver resection can improve the resection rate of the liver tumors, shorten the operation time, reduce intraoperative blood loss, and reduce the occurrence of postoperative complications. It has strong advantages in accurate preoperative evaluation and surgical planning and excellent postoperative management. [40–43].

## Figures and Tables

**Table 1:** Basic characteristics and the bias risk evaluation of the included studies.

Author, year	Disease	Samples (3D/2D)	Age (3D/2D)	Outcomes	NOS
Fang, 2013	Hepatolithiasis	42/56	NA	2), 4), 5)	9
Fang, 2015a	Primary hepatic carcinoma	53/55	53 ± 11/49 ± 11	1), 2), 3), 5), 6)	9
Fang, 2015b	Primary hepatocellular carcinoma	56/64	46.5 ± 13.3/47.5 ± 13.8	1), 2), 3)	8
He, 2015	Echinococcosis of liver	47/59	NA	1), 2), 3)	9
Li, 2017	Primary hepatic carcinoma	24/26	NA	1), 2), 3)	6
Li, 2018	Hepatocellular carcinoma + cholangiocarcinoma + cholangiolithiasis + hepatic parasitosis + cholangiocarcinoma + hemangioma	172/220	42.4 ± 11.4	1), 2), 3), 4), 5), 6)	7
Nakayama, 2017	Hepatocellular carcinoma + intrahepatic cholangiocarcinoma + metastatic liver tumor	120/120	65 (22∼80)/67 (17∼81)	1), 2), 4)	7
Okuda, 2015	Primary cholangiocarcinoma	69/49	66 ± 9/64 ± 11	1), 2)	7
Song, 2020	Primary hepatic carcinoma	59/59	52.31 ± 4.33/51.68 ± 4.29	1), 2), 4), 5), 6)	8
Velayutham, 2016	Colorectal metastasis + hepatocellular carcinoma	40/20	NA	1), 2), 3), 4), 6)	6
Wang, 2021	Liver tumor	34/36	54.18 ± 14.42/59.17 ± 9.24	1), 6)	8
Wei, 2016	Hepatocellular carcinoma	43/31	48 ± 10.0/50.5 ± 10.6	1), 2), 3), 6)	8
Zhang, 2019	Primary hepatic carcinoma	30/34	55.7 ± 11.2/52.5 ± 12.1	1), 2), 6)	8

3D: three-dimensional; 2D: two-dimensional; (1) postoperative complications; (2) operating time; (3) intraoperative blood loss; (4) the incidence of liver failure; (5) the postoperative 1-year recurrence rates; (6) the postoperative 1-year survival rates.

## Data Availability

The data used to support the findings of this study are available from the corresponding author upon request.
